# Trends in opioid prescribing after shoulder arthroplasty in an integrated health system: an analysis across racial and ethnic groups

**DOI:** 10.1016/j.jsea.2026.100031

**Published:** 2026-04-29

**Authors:** David O. Alfaro, Zimin Zhuang, Anna Davis, Heather A. Prentice, Andrew M. Schmidt, Ronald A. Navarro

**Affiliations:** a Kaiser Permanente Bernard J. Tyson School of Medicine, Pasadena, CA, USA; b Kaiser Permanente Southern California, Department of Research and Evaluation, Pasadena, CA, USA; c Center for Effectiveness and Safety Research, Kaiser Permanente Research and Quality Measurement, Pasadena, CA, USA; d Kaiser Permanente Medical Device Surveillance and Assessment, San Diego, CA, USA; e Department of Orthopaedic Surgery, Southern California Permanente Medical Group, Harbor City, CA, USA

**Keywords:** Shoulder arthroplasty, Opioid prescribing, Opioid stewardship, Post-operative pain management, Prescribed opioid reduction, Racial and ethnic disparities

## Abstract

**Background:**

As opioid misuse has increasingly been recognized as a widespread public health threat, healthcare systems have taken steps to decrease prescribing opioids. Given racial and ethnic disparities in pain management have been reported in prior studies, it is important to evaluate whether system-level prescribing changes are implemented equitably across patient populations. Opioids are frequently prescribed to control pain following orthopedic procedures. The purpose of this study was to evaluate trends in opioid prescriptions filled during a period of system-wide opioid reduction initiatives and to determine whether differences in opioid prescriptions filled existed across racial and ethnic groups.

**Methods:**

A retrospective cohort study of opioid prescriptions filled after shoulder arthroplasty from 2016 to 2021 in a closed healthcare system was conducted. The primary outcome was dispensed opioids, quantified as morphine milligram equivalents (MMEs) per calendar day with any supply, in the first month prior to and the 3 months following surgery. The primary explanatory variable was race/ethnicity, with non-Hispanic White as the reference group. Multivariable linear regression was used to determine the trend in the average number of MMEs over the study period.

**Results:**

Over the study period, 1,506 shoulder arthroplasties were performed. In adjusted analyses, average MMEs per supplied day decreased significantly over time, with a large decrease by the final year of the study period (β = −22.79; *P* < .001) relative to the first year. No significant differences in average MMEs per supplied day were observed among the racial and ethnicity groups.

**Conclusion:**

System-wide opioid reduction initiatives were associated with a substantial decline in opioid prescriptions filled after shoulder arthroplasty over the study period. These reductions occurred consistently across racial and ethnic groups, suggesting that prescribing changes were implemented without varying effects across patient populations. Future studies should evaluate patient-reported pain control and the role of multimodal pain management strategies that may have a role in further reducing post-operative opioid prescribing.

Orthopedic surgeons are among the highest prescribers of opioids for pain control.^6^^,^^7^^,^^23^ Studies have reported the efficacy of targeted healthcare system interventions in reducing opioid prescribing.^16^^,^^20^ Kaiser Permanente Southern California recently adopted a multipronged systemic opioid reduction strategy to curb opioid prescriptions in patients undergoing total shoulder arthroplasty (TSA), which led to a significant reduction in morphine milligram equivalents (MMEs) prescribed from 2016 to 2018.^24^ Understanding prescribing patterns during such initiatives is important for evaluating the effectiveness of system-level opioid stewardship efforts.

Prior studies in other healthcare systems have reported racial disparities in TSA, including overall utilization rates, length of hospital stay, mortality, and complications including myocardial infarction, pulmonary embolism, acute renal failure, sepsis, and surgical site infection.^3^^,^^29^ However, a study conducted in an integrated healthcare system found no difference in revision risk and 90-day readmission when comparing racial/ethnic minorities to White patients undergoing TSA.^10^

Prior studies have explored the association between race/ethnicity and opioid prescribing.^12^^,^^14^^,^^22^ Some studies have reported discrepancies in opioid prescribing across racial groups within varied practice settings.^2^^,^^5^^,^^14^^,^^21^ As healthcare systems work to reduce opioid prescribing, it is important to consider the interaction between opioid prescribing and equity across patient populations.^29^ Monitoring prescribing patterns across racial and ethnic groups may help identify whether system-level interventions have unintended consequences that widen disparities within their patient populations, a concept known as a balancing measure within quality improvement.^15^

The purpose of this study was to determine if there were any differences in opioid prescriptions filled after TSA across racial and ethnic groups in a large health system actively engaged in opioid-prescribing reduction strategies. We hypothesized that within our integrated healthcare system opioid prescriptions filled would decrease over time and that no differences would be observed across racial and ethnic groups.

## Methods

### Study design, data source, and study sample

This was a retrospective cohort study conducted in a closed US integrated healthcare system. Data used for the study were obtained from the healthcare system's Shoulder Arthroplasty Registry and integrated electronic health record (EHR) (Epic). A detailed summary of data collection procedures, coverage, and participation rates for the Shoulder Arthroplasty Registry has been previously published.^8^ Briefly, this surveillance tool for all shoulder arthroplasty procedures performed within Kaiser Permanente collects a predefined set of patient, procedure, implant, surgeon, and hospital information using intraoperative forms completed at the point-of-care by the operating surgeon. This information is then supplemented using data from the EHR, administrative claims data, membership data, and mortality records.

This study focused on all-cause primary TSA procedures, including anatomic TSA and reverse TSA, performed in the Southern California region of the healthcare system from January 1, 2016, to December 31, 2021. Patients with revision surgeries, prior surgery on the affected shoulder, prior infection involving the affected shoulder, and same-day bilateral procedures were excluded. The cohort was further restricted to patients who were opioid naive for at least 1 year prior to the perioperative period, defined as having no opioids dispensed in months 2 to 13 before surgery.

### Outcome of interest

The primary outcome evaluated was the number of MMEs prescribed during the 4-month perioperative period surrounding each TSA procedure (1 month presurgery and 3 months postsurgery). The preoperative period was included, as clinicians may place analgesic prescriptions intended for post-operative use during a preoperative visit, while the post-operative period captures ongoing need for pain control following surgery.^27^ To capture the intended dosing of the medications, the total MMEs dispensed to the patient during the perioperative period were divided by the total calendar days with any supply. For patients with multiple prescriptions that overlapped in time, each day was counted once. The resulting outcome variable was average daily MMEs per supplied day.

The analysis included prescriptions written by any provider, as patients may present in a variety of settings with uncontrolled post-operative pain. All medication dispensing data were obtained from the pharmacy module of the integrated EHR, which captures prescriptions filled within the healthcare system's pharmacies. Patients who had no opioids dispensed (MMEs = 0) during the perioperative period were excluded from analysis. Table I lists the typical opioids prescribed during the study period and the MME conversion for each opioid.

**Table I:** Opioids typically prescribed and morphine milligram equivalent conversion factor.

Drug name	Dose	Unit	MME conversion factor
Tramadol	50	mg	0.1
Codeine	30	mg	0.15
Hydrocodone	5	mg	1
Morphine	100	mg	1
Oxycodone	10	mg	1.5
Hydromorphone	2	mg	4
Fentanyl patch	10	mcg/h	2.4

*MME*, morphine milligram equivalent.

### Exposure of interest

The primary exposure variable was race/ethnicity, with non-Hispanic White as the comparison group. Data on race and ethnicity were self-reported during enrollment or when interacting with the healthcare system. Four mutually exclusive categories were included: Asian and Pacific Islander, Black, Hispanic, and non-Hispanic White (henceforth referred to as White). Nine patients with other race and ethnicity categories, including Native American and multiple races/ethnicities, or missing race and ethnicity data were excluded due to low numbers.

### Intervention

Multiple systems-based changes were implemented beginning in 2016 with the goal of reducing opioid prescriptions following TSA within the healthcare system. These changes included the following: 1) elimination of historical physician prescribing presets in the EHR, 2) implementation of a maximum of 20 pills per preset prescription, 3) consistent education and leadership reminders of opioid stewardship using healthcare system-wide emails, and 4) implementation of a state-mandated physician review of individual patient-controlled substance prescription history. Trends in opioid prescriptions by racial and ethnic groups were evaluated across the study period during which these interventions were implemented.

### Covariates

Covariates included for analysis were age, body mass index, sex, the American Society of Anesthesiologists classification, smoking status, household income, surgery type, and indication for surgery. Also included was a covariate for the presence of opioid use-related comorbidities (“opioid-related comorbidities”) as outlined by Rao et al,^26^ which included alcohol abuse, drug abuse, depression, anxiety, psychosis, bipolar disorder, post-traumatic stress disorder, and dementia. Finally, additional covariates were the presence of musculoskeletal pain conditions (history of arthritis, back pain, or limb extremity pain) and chronic pain conditions (history of general chronic pain, kidney stones, gallstones, menstrual pain, migraines, or tension headaches).

### Statistical analysis

In descriptive summary statistics, trends over time in the average MMEs dispensed per supplied day for each race and ethnicity group were reported. Multivariable linear regression models were used to compare average MMEs per supplied day by race and ethnicity; models adjusted for surgery year and the covariates specified above. All analyses were performed using SAS Enterprise Guide version 8.2, and an alpha level of 0.05 was used to determine the statistical significance threshold.

### Institutional review board

This study was approved by the healthcare system's institutional review board prior to its commencement.

## Results

Over the study period, 1,506 TSAs were included in the final study cohort. Of all procedures, 3.1% were performed in Asian patients, 4.4% in Black patients, 18.9% in Hispanic patients, and 73.6% in White patients (Table II). The mean age for the study sample was 70.2 years (standard deviation = 8.7). Most patients were male (55.9%), never smokers (58.4%), had a body mass index ≥25 (43.8%), and had an American Society of Anesthesiologists classification of 1-2 (60.7%). The most common indication for surgery was osteoarthritis (62.7%), and just over half of patients underwent reverse TSA (52%). Most patients did not have comorbidities known to be associated with opioid prescribing following TSA (75.4%) and did not have a history of chronic pain (79.2%). About half of the patients had musculoskeletal pain comorbidities (50.3%).

**Table II:** Characteristics of 1,506 primary shoulder arthroplasty patients by race/ethnicity (2016-2021).

Variable	Asian (n = 46)	Black (n = 67)	Hispanic (n = 285)	White (n = 1,108)
Age in yr				
Mean (SD)	70.9 (7.5)	68.8 (10.8)	70.5 (8.8)	70.2 (8.6)
Range	(57.0-86.0)	(37.0-88.0)	(45.0-94.0)	(39.0-93.0)
Body mass index, kg/m^2^				
<18.5	15 (32.6%)	6 (9.0%)	45 (15.8%)	208 (18.8%)
18.5-25	26 (56.5%)	21 (31.3%)	97 (34.0%)	429 (38.7%)
25-30	5 (10.9%)	23 (34.3%)	92 (32.3%)	307 (27.7%)
≥30	0 (0.0%)	17 (25.4%)	51 (17.9%)	164 (14.8%)
Gender				
Female	18 (39.1%)	29 (43.3%)	150 (52.6%)	466 (42.1%)
Male	28 (60.9%)	38 (56.7%)	135 (47.4%)	642 (57.9%)
ASA classification				
1-2	31 (67.4%)	38 (56.7%)	181 (63.5%)	664 (59.9%)
≥3	15 (32.6%)	28 (41.8%)	89 (31.2%)	319 (28.8%)
Unknown	0 (0.0%)	1 (1.5%)	15 (5.3%)	125 (11.3%)
Smoking status				
Never	28 (60.9%)	40 (59.7%)	193 (67.7%)	618 (55.8%)
Quit	16 (34.8%)	24 (35.8%)	86 (30.2%)	461 (41.6%)
Yes	2 (4.3%)	3 (4.5%)	6 (2.1%)	27 (2.4%)
Unknown	0 (0.0%)	0 (0.0%)	0 (0.0%)	2 (0.2%)
Surgery type				
TSA	21 (45.7%)	32 (47.8%)	99 (34.7%)	571 (51.5%)
Reverse TSA	25 (54.3%)	35 (52.2%)	186 (65.3%)	537 (48.5%)
Indication for surgery				
Osteoarthritis	25 (54.3%)	37 (55.2%)	139 (48.8%)	743 (67.1%)
RCA	15 (32.6%)	25 (37.3%)	127 (44.6%)	306 (27.6%)
Other	6 (13%)	5 (7.5%)	19 (6.7%)	59 (5.3%)
Opioid-related comorbidities				
No	38 (82.6%)	56 (83.6%)	219 (76.8%)	822 (74.2%)
Yes	8 (17.4%)	11 (16.4%)	66 (23.2%)	286 (25.8%)
Musculoskeletal pain				
No	20 (43.5%)	28 (41.8%)	112 (39.3%)	588 (53.1%)
Yes	26 (56.5%)	39 (58.2%)	173 (60.7%)	520 (46.9%)
Chronic pain				
No	40 (87.0%)	51 (76.1%)	230 (80.7%)	872 (78.7%)
Yes	6 (13.0%)	16 (23.9%)	55 (19.3%)	236 (21.3%)
Household income				
Lower 20% ($12,844-$54,888)	5 (10.9%)	15 (22.4%)	84 (29.5%)	145 (13.1%)
20-40% (>$54,888-$73,313)	6 (13.0%)	11 (16.4%)	68 (23.9%)	184 (16.6%)
40-60% (>$73,313-$89,679)	8 (17.4%)	17 (25.4%)	58 (20.4%)	216 (19.5%)
60-80% (>$89,679-$110,373)	13 (28.3%)	15 (22.4%)	48 (16.8%)	267 (24.1%)
Top 20% (>$110,373-$437,289)	14 (30.4%)	8 (11.9%)	27 (9.5%)	290 (26.2%)
Unknown/missing	0 (0.0%)	1 (1.5%)	0 (0.0%)	6 (0.5%)
Yr of surgery				
2016	6 (13.0%)	7 (10.4%)	24 (8.4%)	152 (13.7%)
2017	6 (13.0%)	10 (14.9%)	36 (12.6%)	137 (12.4%)
2018	11 (23.9%)	14 (20.9%)	47 (16.5%)	209 (18.9%)
2019	10 (21.7%)	13 (19.4%)	67 (23.5%)	256 (23.1%)
2020	8 (17.4%)	13 (19.4%)	42 (14.7%)	162 (14.6%)
2021	5 (10.9%)	10 (14.9%)	69 (24.2%)	192 (17.3%)

*TSA*, total shoulder arthroplasty; *ASA*, American Society of Anesthesiologists; *SD*, standard deviation; *RCA*, rotator cuff arthropathy.

**Figure 1:**
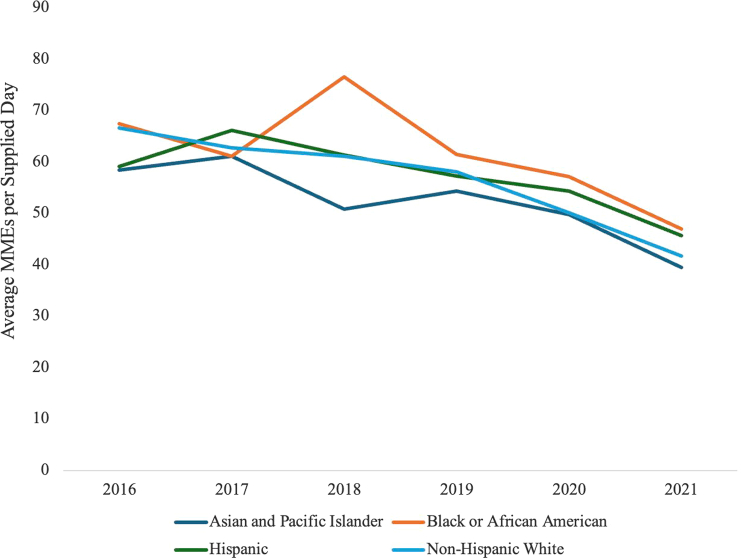
Average MMEs per supplied day in the perioperative period for 1,506 primary shoulder arthroplasty patients across operative years, by race and ethnicity. *MMEs*, morphine milligram equivalents.

Over the study period, average MMEs per supplied day decreased over time. In unadjusted analyses, the average MMEs per supplied day demonstrated a downward trend across all racial and ethnic groups (Fig. 1, Table III). After adjusting for sociodemographic characteristics, surgery type, and clinical factors, average MMEs per supplied day decreased across years (β = −7.36; 95% CI = −11.61 to −3.11; *P* < .001) and an increasingly large effect size over time (Table IV). There were no differences in average MMEs per supplied day when comparing minority racial and ethnic groups to White patients.

**Table III:** Average morphine milligram equivalents per supplied day by year and race/ethnicity following shoulder arthroplasty.

Year	Asian and Pacific Islander	Black or African American	Hispanic	Non-Hispanic White
2016	58.4	67.4	59.1	66.6
2017	61.1	61.1	66.1	62.7
2018	50.9	76.5	61.3	61.1
2019	54.3	61.5	57.3	58.0
2020	49.8	57.1	54.3	50.1
2021	39.4	47.0	45.7	41.7

**Table IV:** Multivariable linear regression results for the association between race/ethnicity and average MMEs per supplied day for 1,506 shoulder arthroplasty patients.

Variable (reference category)	Estimate	95% CI	*P* value
Intercept	61.12	(45.89, 76.34)	<.001
Race/ethnicity (non-Hispanic White)			
Asian and Pacific Islander	−3.77	(−10.82, 3.28)	.29
Black or African American	5.33	(−0.59, 11.24)	.08
Hispanic	1.52	(−1.73, 4.77)	.36
Surgery yr (2016)			
2017	−2.47	(−7.25, 2.32)	.31
2018	−3.73	(−8.14, 0.67)	.10
2019	−7.36	(−11.61, −3.11)	<.001
2020	−14.19	(−18.85, −9.53)	<.001
2021	−22.79	(−27.33, −18.25)	<.001
Age	−0.07	(−0.24, 0.09)	.40
Body mass index	0.34	(0.11, 0.58)	.004
Sex (female)			
Male	1.43	(−1.17, 4.04)	.28
ASA classification (1-2)			
≥3	−1.31	(−4.17, 1.55)	.37
Unknown	0.84	(−3.46, 5.14)	.70
Smoking status (never)			
Quit	−0.19	(−2.71, 2.33)	.88
Yes	−6.80	(−14.61, 1.00)	.09
Unknown	−20.12	(−54.17, 13.93)	.25
Surgery type (TSA)			
Reverse TSA	−1.40	(−5.06, 2.25)	.45
Indication of surgery (osteoarthritis)			
Rotator cuff arthropathy	−0.33	(−4.10, 3.44)	.86
Other	3.04	(−2.56, 8.64)	.29
Opioid-related comorbidities (no)			
Yes	−0.14	(−3.00, 2.72)	.92
Musculoskeletal pain (no)			
Yes	−0.52	(−3.31, 2.27)	.71
Chronic pain (no)			
Yes	−1.03	(−4.09, 2.03)	.51
Household income (top 20%)			
Lower 20% ($12,844-$54,888)	−1.78	(−5.91, 2.35)	.40
20-40% (>$54,888-$73,313)	−0.74	(−4.63, 3.15)	.71
40-60% (>$73,313-$89,679)	2.20	(−1.57, 5.96)	.25
60-80% (>$89,679-$110,373)	−1.13	(−4.73, 2.46)	.54
Unknown/missing	−9.44	(−27.82, 8.95)	.31

*CI*, confidence interval; *MMEs*, morphine milligram equivalents; *TSA*, total shoulder arthroplasty; *ASA*, American Society of Anesthesiologists.

A sensitivity analysis was implemented to test for an interaction between race/ethnicity and surgery year on average MMEs per supplied day. No interaction was observed, suggesting there was not a differential trend over time across racial and ethnic groups. For parsimony and ease of interpretation, these interaction terms were not included in the final model.

## Discussion

This study evaluated opioid prescribing patterns following TSA within a large integrated healthcare system during a period of system-wide opioid reduction initiatives. The principal finding was a substantial decline in opioid prescriptions filled over the study period. Notably, these reductions occurred consistently across racial and ethnic groups, with no significant differences observed in average MMEs per supplied day when compared to White patients. These findings suggest that system-level opioid prescribing reduction initiatives can reduce post-operative opioid prescribing following TSA without introducing differential prescribing patterns across racial and ethnic groups.

Prior studies have found lower opioid prescribing quantities among Black patients relative to White patients across varied practice settings.^1^, ^2^, ^3^^,^^11^^,^^25^ Factors such as inherent, polyfactorial biases, access to care, pain-related attitudes, lack of insurance, and limited availability of resources have been proposed as contributors to these known disparities.^1^^,^^18^^,^^30^^,^^31^ The failure to identify a difference in our study may be due to several of these factors being mitigated within the integrated healthcare system, including insurance coverage and access to care.^10^

Several recent studies examining opioid prescriptions in the post-operative setting for a variety of surgical procedures reported differing prescribing patterns across racial groups.^13^^,^^28^ Romanelli et al conducted a large retrospective study across multiple surgical specialties and found that opioid prescribing patterns by race varied by procedure; for example, Black patients received higher opioid quantities in certain orthopedic procedures such as knee arthroscopy.^28^ Similarly, Herb et al found higher mean MMEs received by Black patients following common general surgery procedures.^13^ The differences observed in these studies compared with our findings illustrate the complexity of racial disparities in healthcare and suggest prescribing patterns may vary across healthcare systems and practice environments.^9^

This study found a sustained downward trend in opioid prescriptions filled following TSA within our health system over the study period. These reductions occurred during the implementation of several system level opioid prescribing reduction initiatives, including changes to EHR prescribing presets, limits on opioid quantities, and physician education regarding opioid prescribing. Implementation of these measures may have contributed to more standardized and conservative prescribing patterns among surgeons performing TSA. These findings are consistent with prior studies reporting that multimodal analgesia strategies and targeted opioid reduction initiatives can reduce post-operative opioid prescribing.^16^^,^^19^ Importantly, these reductions occurred consistently across racial and ethnic groups, suggesting that the system-wide changes were implemented without significant variation in prescribing patterns across patient populations.

Integrated healthcare systems may reduce disparities related to access to care, medication availability, and insurance coverage, which have been identified as potential contributors to differences in opioid prescribing patterns related to race in other healthcare settings.^10^ Our findings suggest integrated systems may be able to help promote more consistent opioid prescribing across diverse patient populations by standardizing prescribing patterns and working to reduce structural barriers to care.

### Limitations

There are some limitations to the present study. First, this study is observational, thus only associations are reported, rather than causation. In our sample of 1,506 primary TSAs, there were relatively few patients in some race and ethnicity groups, which may have limited our ability to identify small differences. Furthermore, we measured opioids filled as opposed to opioids consumed by patients. Direct measurement of opioid consumption is difficult to obtain on a large scale and is susceptible to reporting and recall bias. Therefore, it is possible this study overestimated the amount of opioids ultimately used by patients, and the measure may also represent prescribing patterns of clinicians within the healthcare system. It is also possible that some patients obtained opioid prescriptions outside the integrated healthcare system, which would not be captured in the EHR. However, opioids require a prescription and care outside the healthcare system is typically not covered within the integrated insurance model. Therefore, the likelihood of substantial unobserved prescribing is expected to be low. Another limitation of the study is lack of ancillary data that could potentially confound the need for opioid prescribing, such as patient pain scores and use of adjunct analgesic agents. Further research that focuses on pain control and patient experience may help elucidate whether post-operative pain management is equitable. Recent literature also suggests that use of celecoxib, regional analgesia, and multimodal pain regimens can reduce opioid prescriptions following TSA.^4^^,^^16^^,^^17^^,^^19^ Future studies may examine whether use of such adjuncts differs by race/ethnicity, and, if so, whether they are associated with post-operative opioid prescribing patterns.

## Conclusion

In this cohort, we observed a decline in opioid prescriptions filled following TSA within a large integrated healthcare system during a period of system-wide opioid reduction initiatives. The reduction in opioid prescribing patterns was not observed to differ for minority racial/ethnic groups compared to the White race. These findings suggest that system-level efforts to reduce opioid prescribing can reduce post-operative opioid prescribing while maintaining more equitable prescribing practices across diverse patient populations.

## Disclaimers:

Funding: This study was supported by internal funding from the Kaiser Permanente Bernard J. Tyson School of Medicine.

Conflicts of interest: The authors, their immediate families, and any research foundations with which they are affiliated have not received any financial payments or other benefits from any commercial entity related to the subject of this article.
